# Optimal hyperdimensional representation for learning and cognitive computation

**DOI:** 10.3389/frai.2026.1690492

**Published:** 2026-02-10

**Authors:** Prathyush P. Poduval, Hamza Errahmouni Barkam, Xiangjian Liu, Sanggeon Yun, Yang Ni, Zhuowen Zou, Nathaniel D. Bastian, Mohsen Imani

**Affiliations:** 1 Donald Bren School of Information and Computer Sciences (ICS), University of California, Irvine, Irvine, CA, United States; 2 Purdue University Northwest, Hammond, IN, United States; 3 United States Military Academy, West Point, NY, United States

**Keywords:** brain-inspired learning, cognitive computation, high-dimensional representation, hyperdimensional computing (HDC), neural-symbolic encoding

## Abstract

Hyperdimensional Computing (HDC) is a neurally inspired computing paradigm that leverages lightweight, high-dimensional operations to emulate key brain functions. Recent advances in HDC have primarily targeted two domains: *learning*, where the goal is to extract and generalize patterns for tasks such as classification, and *cognitive computation*, which requires accurate information retrieval for human-like reasoning. Although state-of-the-art HDC methods achieve strong performance in both areas, they lack a principled understanding of the fundamentally different requirements imposed by learning vs. cognition. In particular, existing works provide limited guidance on designing encoding methods that generate optimal hyperdimensional representations for these distinct tasks. In this study, we proposed the first *universal hyperdimensional encoding method* that dynamically adapts to the needs of both learning and cognitive computation. Our approach is based on neural-symbolic techniques that assign random complex hypervectors to atomic bases (e.g., alphabet definitions) and then apply algebraic operations in the high-dimensional *hyperspace* to control the correlation structure among encoded data points. Through theoretical analysis, we show that learning tasks benefit from *correlated* representations to maximize memorization and generalization capacity, whereas cognitive tasks require *orthogonal, highly separable* representations to enable accurate decoding and reasoning. We further derived a separation metric that quantifies this trade-off and validated it empirically across image classification and decoding tasks. Our results demonstrate that tuning the encoder to increase correlation improves classification accuracy from 65% to 95%, while maximizing separation enhances decoding accuracy from 85% to 100%. These findings provide the first systematic framework for designing hyperdimensional encoders that unify learning and cognition under a single, theoretically grounded representation model.

## 1 Introduction

The human brain remains the most sophisticated information processing system known to date, despite decades of breakthroughs in computer science and machine learning. Advances in biological vision, cognitive psychology, and theoretical neuroscience have inspired numerous models that have significantly shaped modern artificial intelligence (AI) (Lindsay, 2020; Indiveri and Horiuchi, 2011; Mitrokhin et al., 2020). Yet, existing AI methods often face fundamental challenges when deployed on real-world platforms, including energy efficiency, robustness under noise, and the ability to generalize with limited data. Thus, bridging the gap between cognitive reasoning and machine learning remains a critical open problem.

Brain-inspired computing approaches attempt to integrate insights from neuroscience into algorithmic frameworks for learning and reasoning. Among these, *Hyperdimensional Computing* (HDC) has emerged as a powerful paradigm that abstracts cognition into computations over high-dimensional distributed representations, or *hypervectors* (Kanerva, 2009, 1988). Unlike conventional representations that store information in localized units, HDC distributes information holographically across all dimensions of a hypervector, enabling inherent robustness to noise, hardware errors, and partial information loss. Moreover, a well-defined set of algebraic operations in the high-dimensional *hyperspace* supports symbolic reasoning and learning in a unified mathematical framework.

Several models fall under the umbrella of HDC (Schlegel et al., 2022; Kleyko et al., 2021, 2016), including Tensor Product Representations, Holographic Reduced Representations (Tay et al., 2019; ; Plate, 1995), Multiply-Add-Permute (Gayler, 1998), Binary Spatter Codes (Kanerva, 1996), and Sparse Binary Distributed Representations (Rachkovskiy et al., 2005). These models differ in how they encode information into hypervectors, yet share common advantages such as distributed storage, associative memory, and robustness to random perturbations. Importantly, unlike spiking neural networks that attempt to mimic neuronal dynamics, HDC focuses on the *representational and computational* aspects of cognition, enabling efficient learning and reasoning in both software and hardware implementations.

Recent efforts have applied HDC to diverse machine learning tasks, including classification (Najafabadi et al., 2016), clustering (Imani et al., 2020), regression (Hernández-Cano et al., 2021), fault detection (Poduval et al., 2021a, 2022a), and face detection (Imani et al., 2022; Poduval et al., 2021b). HDC has also demonstrated potential for cognitive tasks involving symbolic reasoning and graph-based inference (Poduval et al., 2022b), and more broadly as a robust, interpretable representation framework for complex biological and multimodal data (Stock et al., 2024). End-to-end HDC learning frameworks have been developed for event-driven and neuromorphic data (Zou et al., 2022b), and hybrid approaches combining HDC with neural feature extractors have been proposed for federated and edge scenarios (Khaleghi et al., 2024). Comprehensive surveys unify and benchmark the expanding landscape of HDC methods across classification, encoding strategies, and efficiency trade-offs (Kleyko et al., 2021; Vergés et al., 2025). In addition, significant hardware and software advances target energy-efficient deployment, including reconfigurable HDC accelerators (Nayan et al., 2025), heterogeneous programming systems for multi-target compilation (Arbore et al., 2025), and intelligent sensing co-designs for real-time sparse-data processing (Yun et al., 2024). In all such applications, an encoder maps raw data into the hyperspace, after which learning or reasoning proceeds through simple algebraic manipulations over hypervectors. This simplicity opens the door to real-time, low-power, and error-tolerant implementations on emerging hardware platforms.

However, the *design of the encoder* remains a fundamental open question. Current practice relies heavily on empirical choices, with little understanding of how encoder properties influence learning accuracy, reasoning fidelity, or robustness across tasks. This raises several critical questions: *What properties make an HDC encoder suitable for a given application? Do learning and cognitive reasoning tasks impose conflicting requirements on the representation space? Can a single encoder be adapted to meet both?*

**Figure 1:**
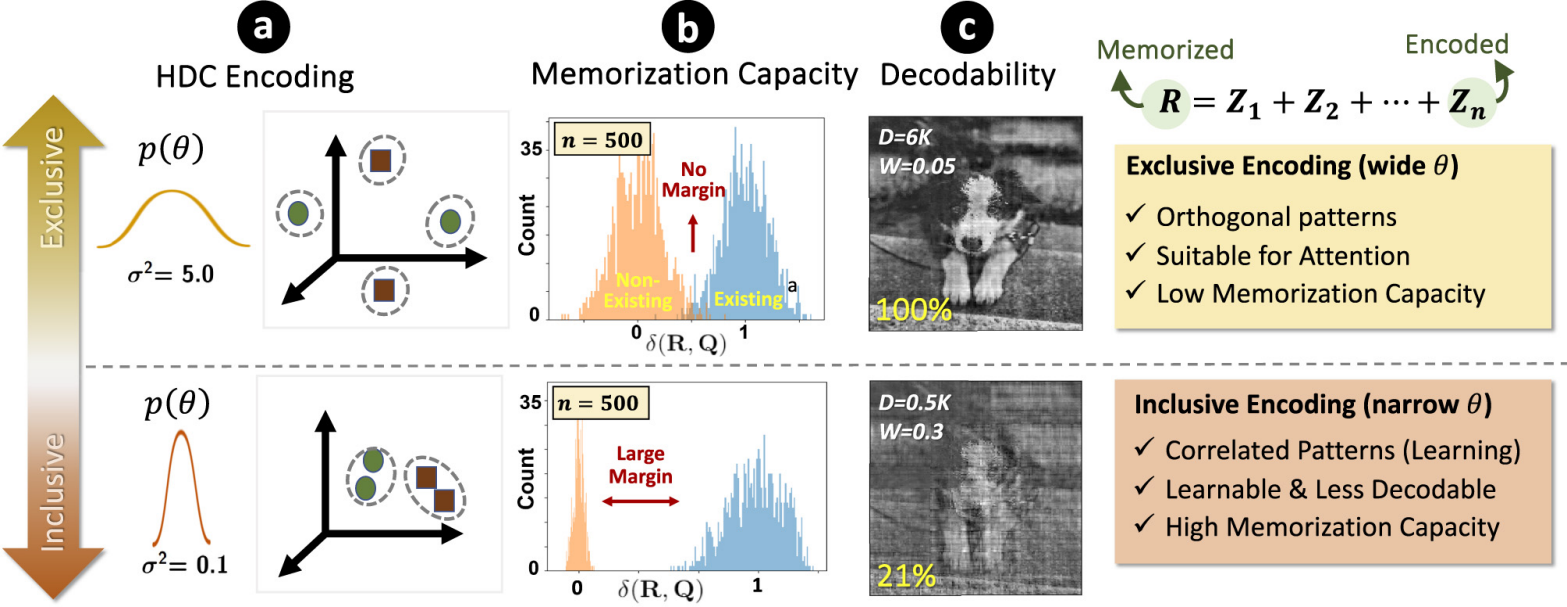
**(a)** Two directions in HDC encoding designs: the correlative approach is suitable for learning since it clusters similar datapoints together, while the exclusive approach is suitable for cognition since each datapoint is encoded individually. **(b)** The Learning capacity is determined by the separation of the signal and noise distribution, which is large in correlative encoding. **(c)** For decodability, the exclusive encoding helps identify each datapoint in the feature space.

Our key insight is that different tasks impose fundamentally different geometric constraints on hyperspace representations. Learning tasks, such as image classification or object detection, require *correlated encodings* that cluster similar inputs to facilitate pattern extraction. In contrast, cognitive tasks, such as question answering or symbolic reasoning, demand *exclusive encodings* that maximize separation between data points to ensure accurate information retrieval and interpretable decoding. Figure 1 illustrates this dichotomy between correlative and exclusive encoder designs.

To address these challenges, we reformulate our contributions as follows.

- **Task-Aware Kernel-Based HDC Encoding**. We instantiate a kernel-based hyperdimensional encoder that is mathematically equivalent to Fractional Power Encoding (FPE) and to Random Fourier Feature constructions in Vector Function Architectures (Plate, 1995; Frady et al., 2022; Rahimi and Recht, 2007). Instead of claiming a new encoding primitive, we focus on how the kernel width *w* controls correlation in hyperspace, thereby imposing opposing requirements for learning vs. cognitive retrieval and factorization.
- **Separation Metrics for Learning and Cognition**. Building on the classical VSA view that nearly orthogonal hypervectors are optimal for associative retrieval (Kanerva, 2009; Plate, 1995; Gayler, 1998), we derive simple, variance-normalized separation metrics that characterize when hypervectors remain decodable (cognition) and when class centroids remain separable (learning) as a function of *w* and dimensionality. These metrics provide an analytical explanation for why learning tasks prefer correlated encodings, whereas retrieval and resonator factorization prefer near-orthogonal encodings.
- **Illustrative Empirical Study and Design Guidelines**. Using a controlled 5 × 5 synthetic pattern and downsampled MNIST (LeCun et al., 1998), we empirically map decoding accuracy, classification accuracy, and resonator convergence as functions of kernel width. While these toy datasets do not aim for state-of-the-art performance, they provide clear evidence that aligns with our analytical predictions and yield practical guidelines for tuning kernel-based HDC encoders. Extending this analysis to richer, continuous-valued, high-dimensional datasets is an important direction for future work.

Our results show that decoding tasks require a separation value of approximately 2–3 for robust performance under noise, whereas learning tasks achieve peak accuracy at lower separation values around 0.8–1.2. Factorization tasks, in which hypervectors must be decomposed into constituent components, demand even higher exclusivity to prevent error propagation during decoding. These findings provide the first unified theoretical and empirical framework for encoder design in HDC.

## 2 Materials and methods

### 2.1 Hyperdimensional computing: an overview

Hyperdimensional Computing (HDC) represents information using large-dimensional vectors, or *hypervectors*, which are nearly orthogonal in the high-dimensional space, also called *hyperspace* (Kanerva, 1998). Computation in HDC proceeds through a small set of algebraic operations on hypervectors. Two key operations form the foundation of HDC: *Bundling* (+), which performs element-wise addition to combine sets of hypervectors, and *Binding* (⊙), which performs element-wise multiplication to encode associations or relationships. Both operations preserve the holographic property of hypervectors, ensuring that information is evenly distributed across all dimensions. Since hypervectors typically have i.i.d. and pseudo-random components, the representations are robust to noise and partial corruption, enabling recovery of information even when some dimensions are missing.

Over the past decade, HDC has been successfully applied to diverse problems, including classification (Kanerva, 2009), activity recognition (Kim et al., 2018), biomedical signal processing (Moin et al., 2021), multimodal sensor fusion (Räsänen and Saarinen, 2015), security (Thapa et al., 2021; Zhang et al., 2021), and distributed sensing (Kleyko et al., 2018). A major advantage of HDC is its ability to learn from few-shot or even one-shot examples, often outperforming support vector machines (SVMs), gradient boosting methods, and convolutional neural networks (CNNs) in settings with limited data (Rahimi et al., 2018; Mitrokhin et al., 2019). Moreover, HDC implementations are highly energy-efficient on embedded processors (Montagna et al., 2018), enabling real-time deployment on low-power hardware platforms.

The design of the *encoder* critically influences both the similarity metric between data points in hyperspace and the degree of correlation or exclusivity in their representations. Figure 1 illustrates two primary encoding regimes. The first is *Correlative Encoding*, which captures shared structure among data points by preserving similarities in hyperspace. This regime is well-suited to learning tasks such as classification, where the objective is to extract patterns from data while maintaining smooth decision boundaries for generalization. The second regime is *Exclusive Encoding*, which maximizes separation between hypervectors to enable accurate decoding of stored information. This regime is essential for cognitive tasks requiring symbolic reasoning, information retrieval, or logical inference.

Therefore, learning tasks benefit from correlated representations that increase capacity and prevent overfitting, whereas cognitive tasks demand orthogonal, highly separated hypervectors for accurate decoding and reasoning. These two contrasting requirements highlight the need for an encoding framework that dynamically tunes the balance between correlation and exclusivity.

### 2.2 Universal neural encoding

**Figure 2:**
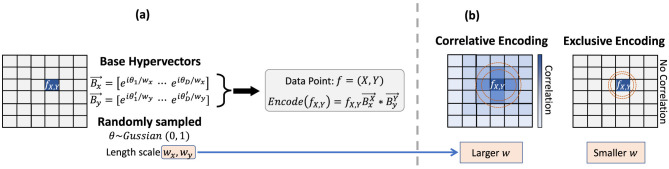
Our universal encoder controls the trade-off between correlation and exclusivity via tunable parameters in the Gaussian kernel. **(a)** Universal HDC Encoding. **(b)** Exclusiveness of Encoding.

Our positional encoder uses complex-valued hypervectors constructed as random phasors. We first sample base vectors ${\vec{\theta }}_{x},{\vec{\theta }}_{y}\sim N{\left(0,1\right)}^{D}$ and define


$$\begin{array}{l} {\vec{B}}_{x}=\exp\left(i\frac{{\vec{\theta }}_{x}}{w_{x}}\right),\;{\vec{B}}_{y}=\exp\left(i\frac{{\vec{\theta }}_{y}}{w_{y}}\right), \end{array}$$ (1)

where *w*_*x*_ and *w*_*y*_ are kernel length scales controlling the correlation structure along the horizontal and vertical axes, respectively. A pixel at the integer coordinate (*X, Y*) is then encoded by binding powers of these bases,


$$\begin{array}{l} {\vec{P}}_{X,Y}={\vec{B}}_{x}^{X}⊙{\vec{B}}_{y}^{Y}, \end{array}$$ (2)

where ⊙ denotes component-wise complex multiplication. In Figure 2, we show the similarity distribution of the kernel generated by the inclusive and correlative encoding, which can be tuned by the scale vectors *w*_*x*_ and *w*_*y*_.

This construction is mathematically equivalent to Fractional Power Encoding (FPE) (; Plate, 1995) and to the Vector Function Architecture proposed by Frady et al. (2022) in which continuous coordinates are represented by exponentiating random base vectors. By Bochner's theorem, exponentiating Gaussian-distributed phases yields a shift-invariant Gaussian kernel whose width is controlled by the scaling parameter (Rahimi and Recht, 2007; Li et al., 2021). In the terminology of HDC, our model instantiates a complex-valued VSA closely related to Fourier Holographic Reduced Representations (FHRR) (Plate, 1995; Kleyko et al., 2021). We therefore do not claim novelty at the level of the encoding primitive; instead, we build on this established mechanism to analyze how the kernel width *w* induces task-dependent trade-offs between learning and cognitive retrieval.

#### 2.2.1 Complex-domain implementation

All hypervectors in this study are represented as complex-valued phasor vectors, following the FHRR-style VSA model (Plate, 1995). Binding is implemented as component-wise complex multiplication, and superposition (bundling) as component-wise addition, optionally followed by normalization. Similarity between two hypervectors is measured by the real part of the normalized complex inner product. This is the same complex-domain framework that underlies the Vector Function Architecture in Frady et al. (2022) and the positional encodings used in resonator-based scene understanding (Renner et al., 2024).

#### 2.2.2 Decoding pipeline

For clarity, we summarize the decoding procedure below.

1. Encode the image *f* as a single hypervector ${\vec{V}}_{f}=\underset{X,Y}{\sum }f_{X,Y}{\vec{P}}_{X,Y}$.
2. For each pixel (*X, Y*), compute a similarity score $s_{X,Y}=\text{Re}(\delta \left({\vec{P}}_{X,Y},{\vec{V}}_{f}\right))$, where δ(·, ·) is the normalized complex inner product and Re(·) denotes the real part.
3. Apply a threshold τ (e.g., the mean score) to obtain a binarized estimate ${\hat{f}}_{X,Y}=𝕀\left[s_{X,Y}\geq \tau \right]$.
4. Optionally, refine the reconstruction by iteratively subtracting the contribution of the current estimate and repeating steps (2)–(3), as described in Section 2.4.

Figures showing continuous similarity distributions (e.g., Figure 4) plot the pre-binarization scores *s*_*X, Y*_ for pixels with ground-truth values of 0 and 1. These histograms are diagnostic tools that show how the kernel width *w* affects the separation between the two score distributions. All reported decoding accuracies are computed after the binarization step by comparing ${\hat{f}}_{X,Y}\in \left\{0,1\right\}$ against the binarized MNIST ground truth *f*_*X, Y*_∈{0, 1} on a per-pixel basis.

### 2.3 Empirical illustration

**Figure 3:**
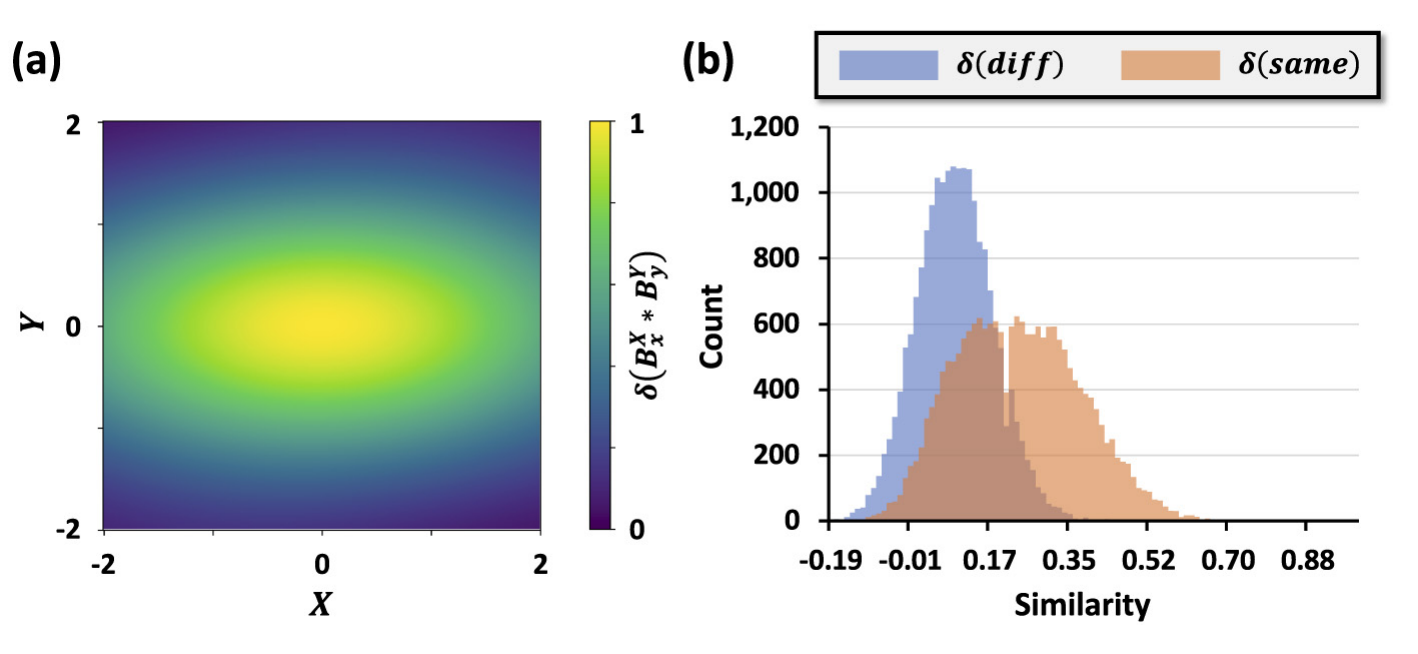
**(a)** The two-dimensional Gaussian kernel for *w*_*x*_ = 2, *w*_*y*_ = 1, with slower decay along the *x*-axis. **(b)** Similarity distributions for images within the same class and across different classes.

The effect of kernel parameters on hyperspace representations is illustrated in Figure 3. Panel (a) shows the two-dimensional Gaussian kernel for *w*_*x*_ = 2 and *w*_*y*_ = 1, with correlations decaying more slowly along the *x*-axis due to the larger length scale. Panel (b) depicts the similarity distributions computed from a synthetic image classification dataset. Images from the same class exhibit high intra-class similarity, while inter-class similarity remains low, demonstrating that the proposed encoder controls separation and correlation in hyperspace via kernel parameters.

### 2.4 Information retrieval for cognition

In cognitive tasks, HDC must accurately retrieve information encoded in hypervectors, which requires careful control of correlations in the representation space. As an illustrative example, consider decoding the original pixel values *f*_*X, Y*_ from the encoded hypervector ${\vec{V}}_{f}$ for an image dataset. Inspired by prior work on HDC-based knowledge extraction and compression (Poduval et al., 2022b), we employ an iterative decoding procedure that refines estimates of *f*_*X, Y*_ by progressively canceling noise contributions in the reconstruction.

Decoding begins by computing an initial estimate of each pixel value based on the similarity between the encoding basis $B_{x}^{X}⊙B_{y}^{Y}$ and the composite hypervector ${\vec{V}}_{f}$. Specifically, the initial estimate is


$$f_{X,Y}^{0}=\text{Binarize}\left(\delta \left(B_{x}^{X}⊙B_{y}^{Y},{\vec{V}}_{f}\right)\right),$$

where the binarization assigns 0 or 1 depending on whether the similarity is below or above its mean value, respectively. This step simplifies the analysis by considering binary features without sacrificing generality, as the approach extends naturally to multi-level quantization.

The initial reconstruction of the encoded vector is then


$${\vec{V}}_{f^{0}}=\underset{X,Y}{\sum }f_{X,Y}^{0}B_{x}^{X}⊙B_{y}^{Y},$$

which is used to approximate noise contributions. The decoding is refined iteratively as


$$f_{X,Y}^{n}=\text{Binarize}\left(\delta \left(B_{x}^{X}⊙B_{y}^{Y},{\vec{V}}_{f}-{\vec{V}}_{f^{n-1}}\right)+f_{X,Y}^{n-1}\right),$$

and repeated until convergence.

A key parameter affecting decoding accuracy is the kernel length scale *w*, which determines correlations among nearby pixels. A small *w* produces nearly orthogonal hypervectors, minimizing interference but losing spatial correlations; a large *w* preserves correlations but introduces overlap between signal and noise. The initial estimate for pixel *f*_*X, Y*_ can be written as


$$\begin{array}{l} f_{X,Y}^{0}=f_{X,Y}+\underset{\text{Noise}\approx N\left(\mu ,\sigma \right)}{\underset{︸}{\underset{X^{\prime }\neq X,Y^{\prime }\neq Y}{\sum }f_{X^{\prime },Y^{\prime }}\delta \left(B_{x}^{X}⊙B_{y}^{Y},B_{x}^{X^{\prime }}⊙B_{y}^{Y^{\prime }}\right)}}, \end{array}$$ (3)

where the second term arises from correlations with all other pixels. Under the assumption of uncorrelated neighboring pixels, the Central Limit Theorem approximates this noise as Gaussian with


$$\begin{array}{r} \mu =\frac{1}{2}\underset{X^{\prime }\neq X,Y^{\prime }\neq Y}{\sum }k\left(\frac{X-X^{\prime }}{w_{x}}\right)k\left(\frac{Y-Y^{\prime }}{w_{y}}\right),\\ \sigma^{2}=\frac{1}{4}\underset{X^{\prime }\neq X,Y^{\prime }\neq Y}{\sum }{\left[k\left(\frac{X-X^{\prime }}{w_{x}}\right)k\left(\frac{Y-Y^{\prime }}{w_{y}}\right)\right]}^{2}, \end{array}$$

where *k*(·) denotes the Gaussian kernel.

**Figure 4:**
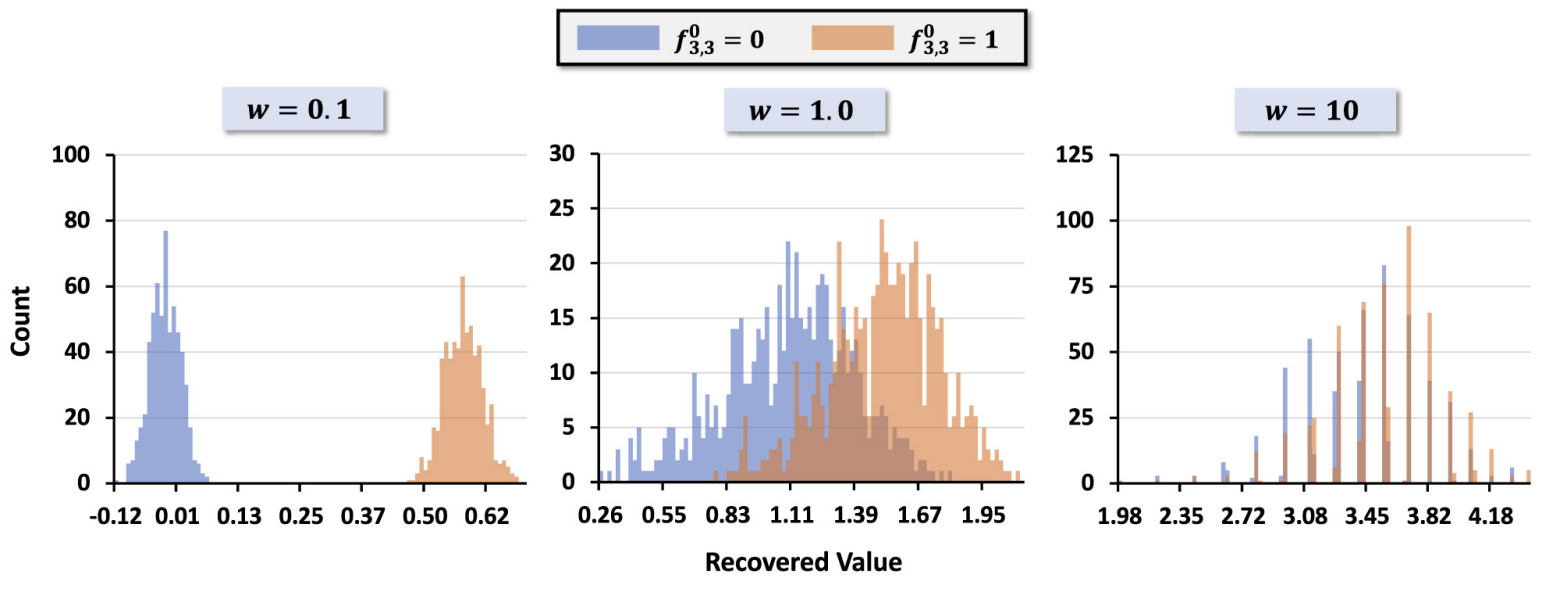
Distributions of the similarity scores used in decoding for different kernel widths *w*. For each *w*, we plot the similarity between the positional basis hypervector and the composite image hypervector for pixels whose ground-truth value is 0 (blue) or 1 (orange) in the synthetic 5 × 5 dataset. These histograms are computed before binarization and are used to visualize how *w* affects the separation between the two score distributions.

**Figure 5:**
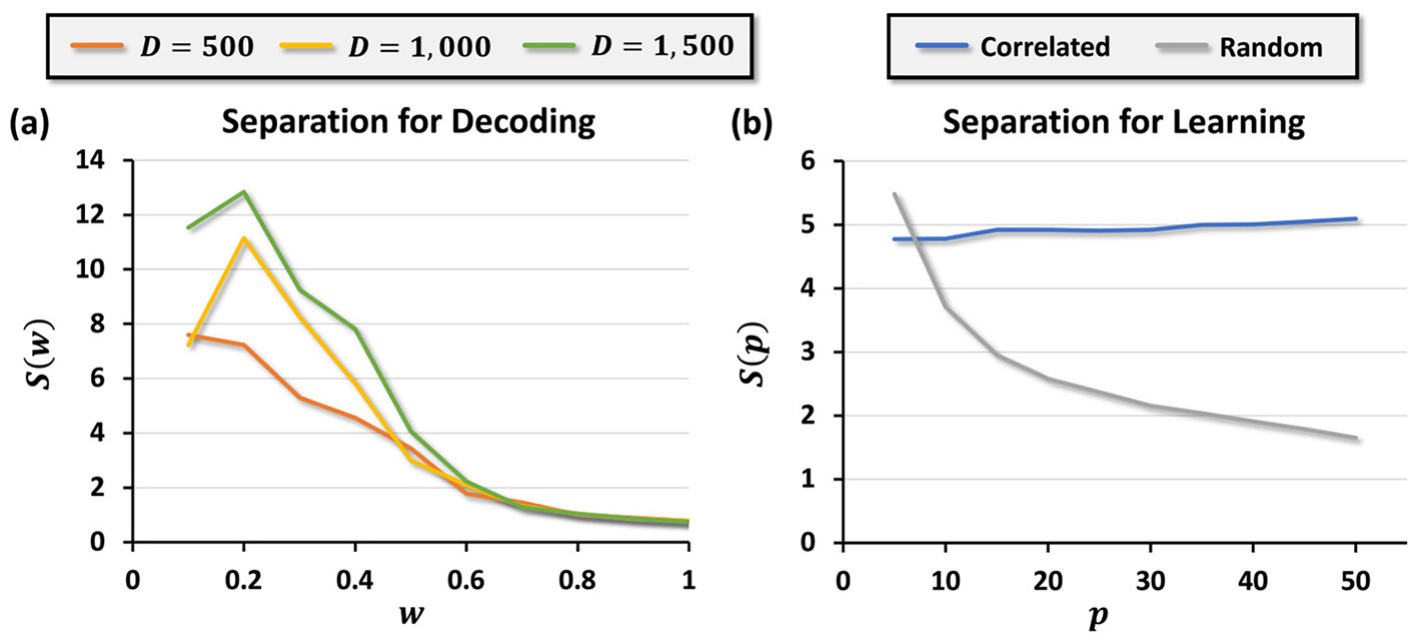
**(a)** Separation *s*(*w*) for different values of dimension *D* in the case of decoding and **(b)** *s*(*p*) Separation as a function of number of learning data points for the case of memorization.

To illustrate, we consider a 5 × 5 image with the center pixel set to 0 or 1, while all other pixels are randomly generated. Figure 4 shows the distributions of the recovered center pixel $f_{3,3}^{0}$ for different kernel widths *w* = *w*_*x*_ = *w*_*y*_. Small *w* values produce well-separated distributions for the two cases, whereas larger *w* values produce significant overlap. A practical criterion for reliable decoding is that the difference between the distribution means must exceed the sum of their standard deviations. We formalize this as a *separation metric*


$$\begin{array}{l} s=\frac{\mu_{2}-\mu_{1}}{\sigma_{1}+\sigma_{2}}, \end{array}$$ (4)

where μ_*i*_ and σ_*i*_ denote the mean and standard deviation for the two cases. Figure 5a plots *s*(*w*) across various hypervector dimensions *D*, showing that separation decreases as *w* increases. This provides a principled way to select *w* for decoding tasks: a small *w* ensures robust information retrieval by maximizing *s*(*w*).

In the decoding setting, separability is evaluated using one-dimensional similarity scores: for each pixel, we compared the scalar similarity between its positional basis hypervector and the composite image hypervector under the two ground-truth cases *f*_*X, Y*_ = 0 vs. *f*_*X, Y*_ = 1. For such one-dimensional distributions, we adopt a symmetric, variance-normalized separation index closely related to the classical signal-detection measure *d*′ (Macmillan and Creelman, 2004). This metric directly reflects the degree of overlap between the two score distributions and is easy to interpret as an effect size. Conversely, multivariate measures such as the Mahalanobis distance are unnecessary here: they reduce simple variance-normalized differences in one dimension and would require estimating covariance matrices without providing additional insight. Thus, our choice balances interpretability and analytical convenience while remaining consistent with standard practice in detection-theoretic analyses.

### 2.5 HDC memorization and pattern extraction for learning

While decoding tasks require orthogonal representations, learning tasks, such as classification, benefit from correlated encodings that capture shared structures across samples. Consider *p* training samples encoded as hypervectors $\left\{{\vec{H}}_{1},{\vec{H}}_{2},\ldots ,{\vec{H}}_{p}\right\}$ and stored via bundling into a class hypervector


$$\vec{C}=\overset{p}{\underset{i=1}{\sum }}{\vec{H}}_{i}.$$

A query hypervector $\vec{Q}$ belongs to the class if its similarity with $\vec{C}$ significantly exceeds its similarity to hypervectors from other classes.

By the Central Limit Theorem, the similarity between $\vec{Q}$ and its correct-class hypervector follows


$$\delta \left(\vec{Q},\vec{C}\right)\sim N\left(p\mu_{1},\sqrt{p}\sigma_{1}\right),$$

while the similarity with an incorrect class hypervector follows


$$\delta \left(\vec{Q},{\vec{C}}^{\prime }\right)\sim N\left(p\mu_{12},\sqrt{p}\sigma_{12}\right),$$

where μ_1_>μ_12_ since intra-class similarities exceed inter-class similarities. The separation between these distributions is then


$$\begin{array}{l} s=\frac{p\mu_{1}-p\mu_{12}}{\sqrt{p}\sigma_{1}+\sqrt{p}\sigma_{12}}=\sqrt{p}\frac{\mu_{1}-\mu_{12}}{\sigma_{1}+\sigma_{12}}. \end{array}$$ (5)

This expression shows that separation improves as $\sqrt{p}$, implying that adding more training samples enhances learning capacity when correlations are maintained.

**Figure 6:**
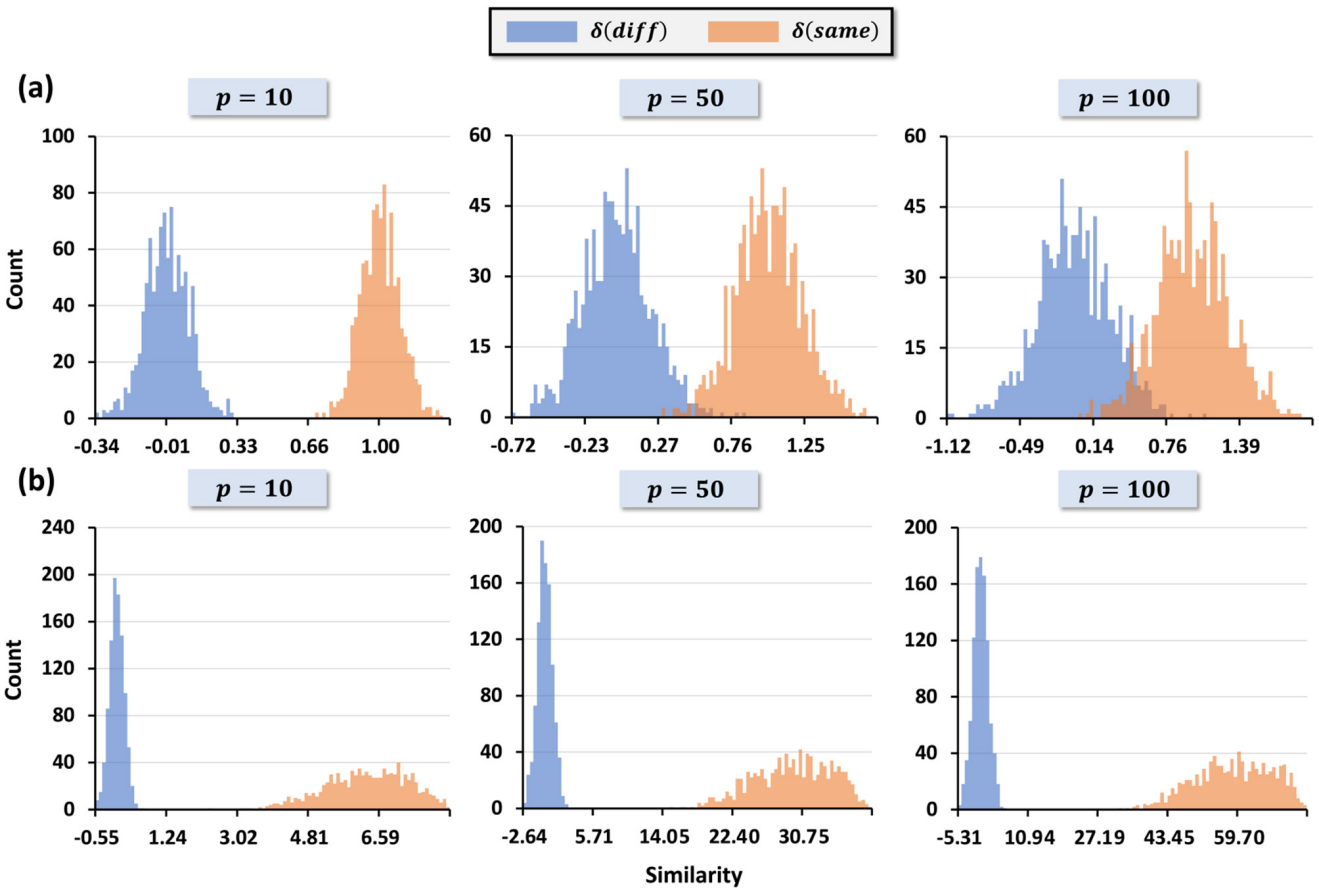
The signal and noise distribution for **(a)** exclusive encoding suitable for cognition and **(b)** correlated encoding for different values of *p*, suitable for learning. The results were calculated for *D* = 1500.

Figures 5b, 6 empirically validate this result. Random encodings cause separation to collapse as *p* increases, whereas correlated encodings maintain high separation, enabling robust classification. Specifically, Figure 6a shows the signal and noise distributions for random encoding, where overlap grows with *p*, while Figure 6b shows correlated encoding preserving distributional separation even as *p* increases.

Together, these results reveal a fundamental trade-off: learning tasks require correlated encodings to exploit shared structure among samples, whereas decoding tasks demand orthogonal encodings for accurate information retrieval. The kernel parameters *w*_*x*_, *w*_*y*_ thus provide a tunable mechanism for balancing these competing requirements.

To make the analysis concrete, we begin with a simple synthetic 5 × 5 dataset. Each image has a designated central pixel at (3, 3) whose binary value determines the class label, while all other pixels are drawn independently from a Bernoulli(0.5) distribution. This construction ensures that class discrimination depends only on the central pixel, allowing the effect of the encoder and its correlation structure to be studied in isolation from more complex spatial cues.

For both the synthetic dataset and MNIST, we use a centroid-based classifier in hyperspace. Given the encoded training hypervectors $\left\{{\vec{H}}_{i}\right\}$ for a class *c*, we form a class centroid


$${\vec{C}}_{c}=\underset{i\in D_{c}}{\sum }{\vec{H}}_{i},$$

where $D_{c}$ indexes the training samples of class *c*. A test hypervector $\vec{Q}$ is then assigned to the class whose centroid has maximum cosine similarity:


$$ŷ\left(\vec{Q}\right)=\arg\underset{c}{\max}\delta \left(\vec{Q},{\vec{C}}_{c}\right).$$

Unless otherwise stated, we sweep the kernel width *w* over a predefined range and report the resulting classification accuracies as functions of (*D, w*). The heuristic in Section 3.2, which relates *w* to empirical correlation lengths in the data, is used only as an intuitive guideline for selecting reasonable scales; our primary objective is to characterize how changes in *w* affect learnability rather than to optimize *w* for a particular benchmark.

### 2.6 Memorizing associations

Hyperdimensional Computing (HDC) encodes associations in a distributed and noise-robust manner using the *binding* operation. If $\vec{A}$ and $\vec{B}$ are two nearly orthogonal bipolar hypervectors, their binding $\vec{S}=\vec{A}⊙\vec{B}$ produces a hypervector nearly orthogonal to both $\vec{A}$ and $\vec{B}$, owing to the inherent randomness in high dimensions. This property enables HDC to represent complex structures such as key–value pairs (Rachkovskij, 2015; Plate, 1995), sequences (Zou et al., 2022a; Poduval et al., 2021c), and multi-attribute data (Frady et al., 2020), in a single holographic representation.

For example, consider the task of memorizing an object along with the location, time, and size at which it was observed. Each object is represented by a hypervector sampled from a discrete codebook $O=\left\{{\vec{O}}_{1},\ldots ,{\vec{O}}_{n}\right\}$, where each ${\vec{O}}_{i}$ corresponds to a distinct entity, such as a ball, cat, or dog. The location, time, and size are continuous-valued attributes, which we encode using the kernel-based method described in earlier sections. Specifically, we assign random base hypervectors (*B*_*X*_, *B*_*T*_, *B*_*S*_) to the spatial, temporal, and size dimensions, with each base vector generated as


$${\vec{B}}_{i}=e^{i{\vec{\theta }}_{i}/w_{i}},\;{\vec{\theta }}_{i}\sim N{\left(0,1\right)}^{D},$$

with *w*_*i*_ controlling the correlation length scale along dimension *i*∈{*X, T, S*}. A continuous value (*x, t, s*) for position, time, and size is encoded as $\left({\vec{B}}_{X}^{x},{\vec{B}}_{T}^{t},{\vec{B}}_{S}^{s}\right)$. The association of object *O*_*i*_ with its features (*x, t, s*) is then stored in the single hypervector


$$\vec{H}={\vec{O}}_{i}⊙{\vec{B}}_{X}^{x}⊙{\vec{B}}_{T}^{t}⊙{\vec{B}}_{S}^{s}.$$

Given a hypervector $\vec{H}$, recovering its factors $\left({\vec{O}}_{i},{\vec{B}}_{X}^{x},{\vec{B}}_{T}^{t},{\vec{B}}_{S}^{s}\right)$ is a nontrivial problem because binding is a lossy superposition in high dimensions. The state-of-the-art approach for this factorization is the *resonator network* (Frady et al., 2020), a recurrent iterative algorithm that refines factor estimates over successive iterations by canceling noise contributions.

Assume that position, time, and size values are drawn from discrete sets {*x*_1_, …, *x*_*n*_}, {*t*_1_, …, *t*_*n*_}, and {*s*_1_, …, *s*_*n*_}. At iteration (*n*−1), let the current guesses for the factors be $\left\{{\vec{G}}_{A,n-1}\right\}$, with *A*∈{*O, X, T, S*}. At the next iteration, the guess for factor *A* is updated by unbinding the current estimates of all other factors from $\vec{H}$, then projecting onto the subspace spanned by the corresponding codebook:


$$\begin{array}{l} {\vec{G}}_{A,n}={\left[\text{M}\right]}_{A}\left(\vec{H}⊙\underset{i\neq A}{\prod }{\vec{G}}_{i,n-1}\right), \end{array}$$ (6)

where [M]_*A*_ denotes the projection operator onto the subspace associated with factor *A*.

Intuitively, each iteration eliminates contributions from all factors except *A*, yielding progressively cleaner estimates as noise terms cancel out. For sufficiently large hypervector dimension *D* and small correlation length scales *w*_*i*_, the resonator network converges to the correct factorization. However, when correlations are high (large *w*_*i*_), the representations of different factors overlap significantly, and the network may converge to spurious solutions. A full theoretical analysis of this nonlinear recurrent system remains challenging; thus, we rely on experimental evaluation to study convergence under varying correlation parameters.

## 3 Experimental results

This section presents a comprehensive empirical analysis of the proposed universal hyperdimensional encoding across cognitive information retrieval (decoding), statistical learning (classification), and factorization. The focus is on how the kernel length scales (*w*_*x*_, *w*_*y*_) and the hypervector dimension *D* modulate the correlation structure in hyperspace and, in turn, performance. All experiments are implemented in PyTorch on an Intel Core i7–12700K platform.

### 3.1 Experimental setup

We conducted experiments to examine how encoder settings influence HDC performance for both learning and cognitive information retrieval. We selected the MNIST handwritten digits as the primary benchmark. The original dataset comprises 28 × 28 grayscale images with intensities in [0, 1]. For a controlled analysis without loss of generality, we cropped and downsampled to 13 × 13 and then binarized the pixel values to {0, 1}. This preserves salient structure while simplifying the decoding analysis and the computations of the separation metric introduced earlier. Unless otherwise noted, we vary *D* and (*w*_*x*_, *w*_*y*_) systematically to expose the correlation–orthogonality trade-off predicted by our theory.

Our empirical evaluation is intentionally based on a small synthetic 5 × 5 example and on the MNIST handwritten digits. The goal of this work is not to compete with state-of-the-art deep networks on challenging benchmarks but to provide a controlled testbed for analyzing how the kernel length scales (*w*_*x*_, *w*_*y*_) govern the trade-off between correlation and orthogonality in hyperspace for learning vs. cognition.

MNIST is widely regarded as a saturated benchmark for modern machine learning: convolutional neural networks routinely exceed 99% test accuracy, and even shallow classical models often reach around 97% accuracy. Thus, several authors have argued that MNIST is “too easy” for evaluating new architectures and have proposed more challenging variants such as EMNIST, Fashion-MNIST, and Oracle-MNIST (Wang and Deng, 2024). Conversely, the HDC literature often uses (binarized or downsampled) MNIST and Fashion-MNIST precisely because their simplicity and standardization allow performance changes to be attributed directly to encoding choices rather than to dataset intricacies (Smets et al., 2024). Our experiments follow this latter tradition: we deliberately choose a simple, well-understood dataset to expose how varying *w*_*x*_ and *w*_*y*_ affects decodability, classification accuracy, and the separation metrics derived in Sections 2.5 and 2.6.

Although we binarized MNIST for analytic clarity in the decoding analysis, the encoder in Equation 1 is linear in the pixel intensity *f*_*X, Y*_, and all theoretical results depend only on the correlations between basis hypervectors, not on the discretization of *f*_*X, Y*_. Hence, the same formulation applies directly to real-valued inputs *f*_*X, Y*_∈ℝ and to higher-resolution images by replacing the binary weights in the superposition with continuous-valued intensities. Moreover, MNIST still exhibits non-trivial local spatial correlations (e.g., strokes, loops, and thickness variations), so sweeping (*w*_*x*_, *w*_*y*_) already reveals how aligning or misaligning the positional code with these correlations affects decoding and learning.

We acknowledge that extending the empirical study to more complex or strongly correlated datasets (e.g., Fashion-MNIST, Oracle-MNIST, or natural images) would further substantiate the generality of the proposed framework. To keep the present manuscript focused on the fundamental representational trade-offs, we leave these extensions for future study and explicitly highlight this as a limitation in the Conclusion.

### 3.2 Encoding: learning vs. cognition

The kernel length scales (*w*_*x*_, *w*_*y*_) determine how quickly positional basis vectors decorrelate with distance in the image: small values induce near-orthogonality across nearby locations, whereas large values preserve similarity over broader neighborhoods. From the analysis in Sections 2.6 and 2.5, we expect decoding and factorization (cognition) to favor small (*w*_*x*_, *w*_*y*_) while learning to favor intermediate values that preserve intra-class correlations without collapsing inter-class structure.

To connect these parameters to the empirical statistics of a dataset, we consider horizontal and vertical co-activation probabilities. For each pixel position (*X, Y*) we estimate


$$\begin{array}{l} p^{1}\left(x,X,Y\right)=\mathbb{P}(f_{x,Y}=1\wedge f_{X,Y}=1),\\ p^{2}\left(y,X,Y\right)=\mathbb{P}(f_{X,y}=1\wedge f_{X,Y}=1), \end{array}$$

from the training set and compute average correlation lengths


$$l_{x}=\langle |x-X|\rangle_{p^{1}},\;l_{y}=\langle |y-Y|\rangle_{p^{2}},$$

as expected horizontal and vertical distances over which pixels tend to co-activate. These lengths provide an intuitive heuristic for choosing kernel scales,


$$w_{x}\approx l_{x},\;w_{y}\approx l_{y},$$

so that the positional code respects the dominant spatial correlations present in the data.

**Figure 7:**
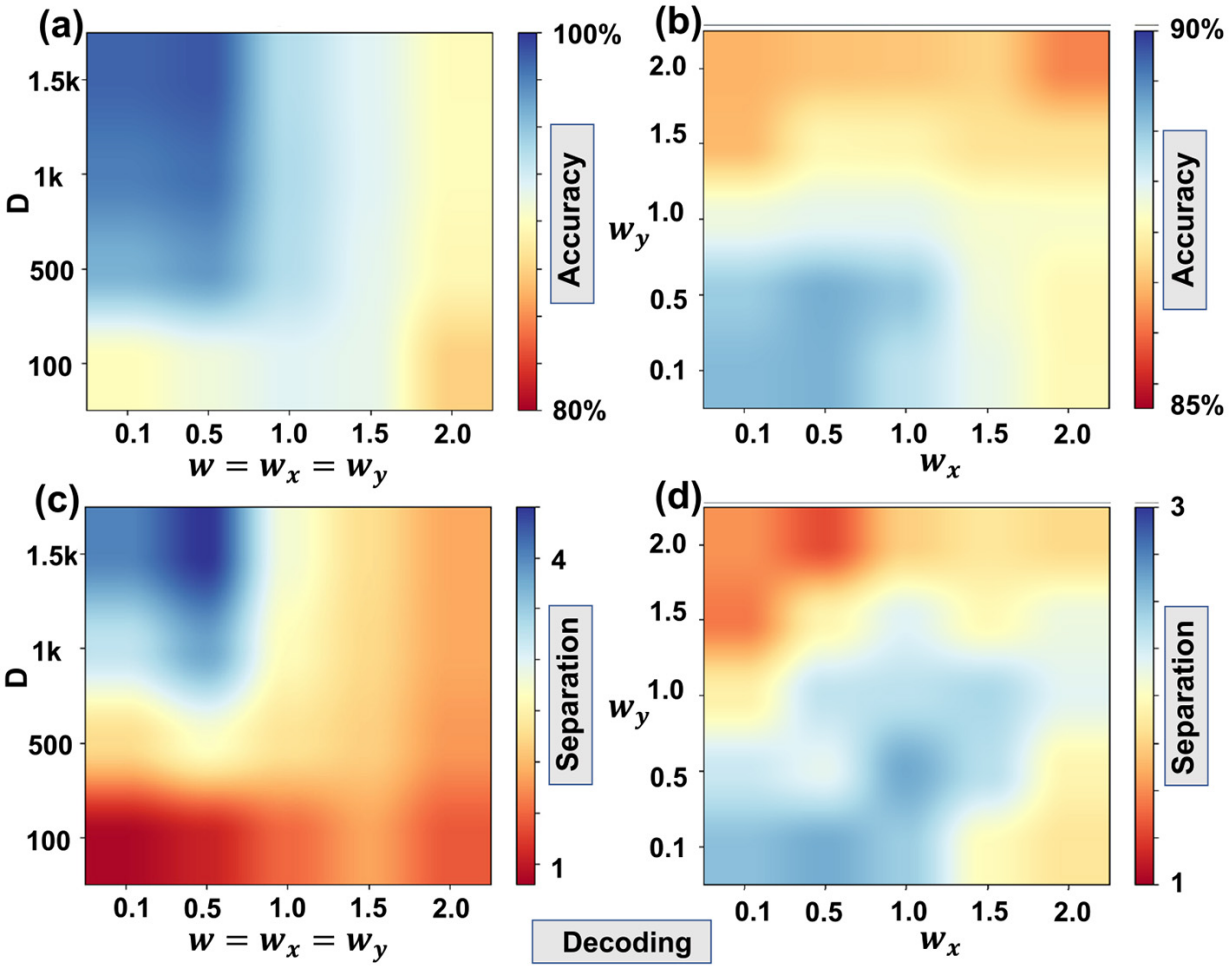
The decoding accuracy as **(a)** function of *D* and *w* **(b)** function of *w*_*x*_ and *w*_*y*_; the decoding separation as **(c)** function of *D* and *w* **(d)** function of *w*_*x*_ and *w*_*y*_.

**Figure 8:**
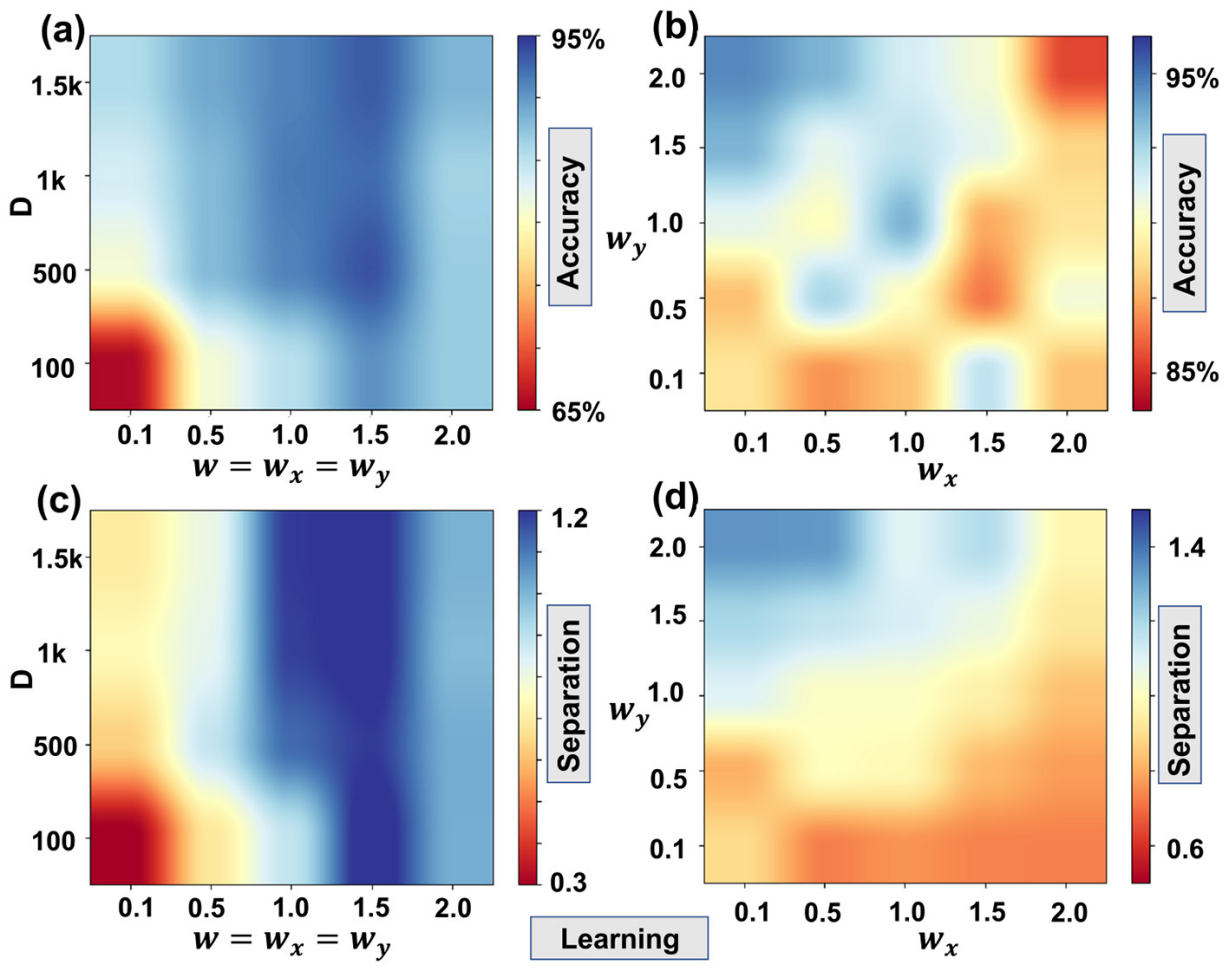
The classification accuracy as **(a)** a function of *D* and *w* **(b)** a function of *w*_*x*_ and *w*_*y*_; the classification separation as **(c)** a function of *D* and *w* **(d)** a function of *w*_*x*_ and *w*_*y*_. The small-*w* regime corresponds to nearly random/orthogonal positional encodings and serves as the baseline case. As *w* increases from this baseline, moderate correlations between nearby positions improve intra-class clustering and thus accuracy; for large *w*, over-correlation causes different classes to become less separable and accuracy decreases.

We emphasized that in this study, we used this rule only as a guideline for selecting a reasonable range of (*w*_*x*_, *w*_*y*_) values. All decoding and learning experiments sweep over a grid of (*w*_*x*_, *w*_*y*_) and report performance as a function of these parameters, rather than relying exclusively on the correlation-length estimate. A fully automated, data-driven procedure for selecting the optimal (*w*_*x*_, *w*_*y*_) across tasks and datasets is an important direction for future study, but it lies beyond the scope of the present study. Here, our primary objective is to show how varying (*w*_*x*_, *w*_*y*_) mediates the trade-off between learning and cognition, and to connect this trade-off qualitatively to the underlying spatial correlation structure of the data. Tables 1, 2 Show the data results corresponding to Figures 7, 8 respectively

**Table 1:** Table corresponding to Figure 7.

**(a)**					
*D*\*w*	0.1	0.5	1	1.5	2
1, 500	0.98	0.99	0.94	0.92	0.90
1, 000	0.98	0.98	0.94	0.92	0.90
500	0.96	0.97	0.94	0.92	0.90
100	0.90	0.91	0.92	0.92	0.87
**(b)**					
*w*_*y*_\*w*_*x*_	0.1	0.5	1	1.5	2
2	0.90	0.90	0.90	0.91	0.89
1.5	0.90	0.92	0.92	0.91	0.91
1	0.93	0.94	0.94	0.93	0.93
0.5	0.96	0.97	0.96	0.93	0.92
0.1	0.96	0.97	0.95	0.94	0.92
**(c)**					
*D*\*w*	0.1	0.5	1	1.5	2
1, 500	4.02	4.51	2.78	2.28	1.89
1, 000	3.23	3.80	2.62	2.24	1.90
500	2.26	2.69	2.33	2.16	1.80
100	0.87	1.07	1.52	1.86	1.43
**(d)**					
*w*_*y*_\*w*_*x*_	0.1	0.5	1	1.5	2
2	1.52	1.30	1.74	1.85	1.77
1.5	1.44	1.94	2.19	1.97	2.12
1	1.91	2.31	2.33	2.39	2.17
0.5	2.28	2.16	2.61	2.35	1.94
0.1	2.51	2.60	2.45	1.98	1.84

The decoding accuracy as **(a)** function of *D* and *w* **(b)** function of *w*_*x*_ and *w*_*y*_; the decoding separation as **(c)** function of *D* and *w* **(d)** function of *w*_*x*_ and *w*_*y*_.

**Table 2:** Table corresponding to Figure 8.

**(a)**					
*D*\*w*	0.1	0.5	1	1.5	2
1, 500	0.86	0.89	0.91	0.93	0.88
1, 000	0.84	0.88	0.92	0.92	0.86
500	0.81	0.88	0.91	0.94	0.87
100	0.66	0.81	0.85	0.90	0.87
**(b)**					
*w*_*y*_\*w*_*x*_	0.1	0.5	1	1.5	2
2	0.94	0.93	0.91	0.90	0.86
1.5	0.93	0.91	0.92	0.91	0.89
1	0.91	0.90	0.93	0.88	0.89
0.5	0.88	0.92	0.90	0.87	0.90
0.1	0.89	0.88	0.88	0.92	0.88
**(c)**					
*D*\*w*	0.1	0.5	1	1.5	2
1, 500	0.69	0.81	1.19	1.22	1.01
1, 000	0.74	0.82	1.18	1.23	0.99
500	0.63	0.89	1.12	1.19	1.02
100	0.29	0.68	0.89	1.25	1.02
**(d)**					
*w*_*y*_\*w*_*x*_	0.1	0.5	1	1.5	2
2	1.34	1.34	1.09	1.18	0.98
1.5	1.20	1.16	1.12	1.06	0.93
1	1.10	1.01	1.01	0.96	0.84
0.5	0.81	1.00	0.98	0.82	0.78
0.1	0.89	0.73	0.76	0.74	0.73

The classification accuracy as **(a)** function of *D* and *w* **(b)** function of *w*_*x*_ and *w*_*y*_; the classification separation as **(c)** function of *D* and *w* **(d)** function of *w*_*x*_ and *w*_*y*_.

### 3.3 Cognition: HDC decodability

In all decoding experiments, accuracy is measured as *per-pixel reconstruction accuracy*. Given a binarized ground-truth image *f* with pixels *f*_*X, Y*_∈{0, 1} and its decoded estimate $\hat{f}$ obtained from the hypervector, we define


$$\text{Ac}{\text{c}}_{\text{dec}}=\frac{1}{\left|\text{Ω}\right|}\underset{\left(X,Y\right)\in \text{Ω}}{\sum }𝕀\left[{\hat{f}}_{X,Y}=f_{X,Y}\right],$$

where Ω is the set of pixel coordinates and *𝕀*[·] denotes the indicator function. To compute ${\hat{f}}_{X,Y}$, we first obtain a continuous similarity score *s*_*X, Y*_ between the positional basis hypervector ${\vec{P}}_{X,Y}$ and the composite image hypervector, then apply a fixed threshold τ (e.g., the mean score) to produce a binary estimate ${\hat{f}}_{X,Y}=𝕀\left[s_{X,Y}\geq \tau \right]$. Figures that show continuous similarity distributions (such as Figure 4) plot the pre-binarization scores *s*_*X, Y*_ for diagnostic purposes, whereas all reported decoding accuracies are computed from the binarized reconstructions ${\hat{f}}_{X,Y}$ and compared with the binarized MNIST ground truth.

We first quantified decodability by reconstructing the binarized image from its hypervector. Figure 7a shows decoding accuracy as a joint function of *D* and an isotropic kernel width *w* with *w*_*x*_ = *w*_*y*_ = *w*. Accuracy is high for small *w*, reflecting that near-orthogonal positional codes keep cross-terms in (3) small; it ranges from 85% at the lowest to 100% at the highest settings we tested. As *w* increases, neighboring positions become correlated and $\delta \left(B_{x}^{X}⊙B_{y}^{Y},B_{x}^{X^{\prime }}⊙B_{y}^{Y^{\prime }}\right)$ grows for (*X*′, *Y*′)≠(*X, Y*), thereby elevating the effective noise. A clear degradation boundary emerges around *w*≈1.5, and the drop becomes sharper as *w* grows beyond roughly 0.75–1.5, consistent with the distributional overlap analysis in Figure 4 and the separation definition in (4). Larger *D* mitigates projection noise and stabilizes accuracy, consistent with concentration effects in high dimensions.

Figure 7b separates horizontal and vertical effects at fixed *D* = 500. We observed boundaries near *w*_*y*_≈1.0 and *w*_*x*_≈1.5, beyond which decodability deteriorates. This anisotropy is consistent with MNIST digit morphology: vertical strokes are more regular, so increasing *w*_*y*_ is initially less harmful than increasing *w*_*x*_. The corresponding separation maps in Figures 7c, d track these transitions: as *w* (or one of *w*_*x*_, *w*_*y*_) increases, the signal and noise distributions approach each other, and the separation in (4) declines, correlating with reduced decoding accuracy in the mid-accuracy regime. Near saturation (>85% accuracy), separation becomes less predictive because errors are dominated by a small number of ambiguous pixels.

### 3.4 HDC Learnability

We next evaluated classification from hypervectors. Figure 8a reports accuracy vs. (*D, w*) with *w*_*x*_ = *w*_*y*_ = *w*. Unlike decoding, learning exhibits a non-monotonic dependence on *w*. When *w* is too small, each sample is mapped to an almost independent code, intra-class similarity is suppressed, and the classifier cannot pool evidence; when *w* is too large, inter-class codes overlap, and the decision boundary collapses. Peak accuracy occurs at an intermediate value around *w*≈0.5 for sufficiently large dimensions (e.g., *D*≳1.5k), where correlations are strong enough to capture shared structure without eroding class separability.

Figure 8b probes anisotropy at fixed *D*. Increasing *w*_*x*_ generally improves accuracy more than increasing *w*_*y*_, reflecting that MNIST class distinctions rely more on horizontal variation (e.g., left–right strokes and loops). The separation plots in Figures 8c, d corroborate these accuracy trends: class-level separation is maximal in the same intermediate-*w* band that yields the best generalization, and it decreases on either side as encodings become either too exclusive (under-smoothing) or too inclusive (over-smoothing). These observations are consistent with the capacity analysis in Section 2.5, where the separation between the class-similarity distributions scales as $\sqrt{p}\left(\mu_{1}-\mu_{12}\right)/\left(\sigma_{1}+\sigma_{12}\right)$ and is optimized when the encoder preserves intra-class correlations while keeping cross-class similarity low.

### 3.5 HDC factorization problem

**Figure 9:**
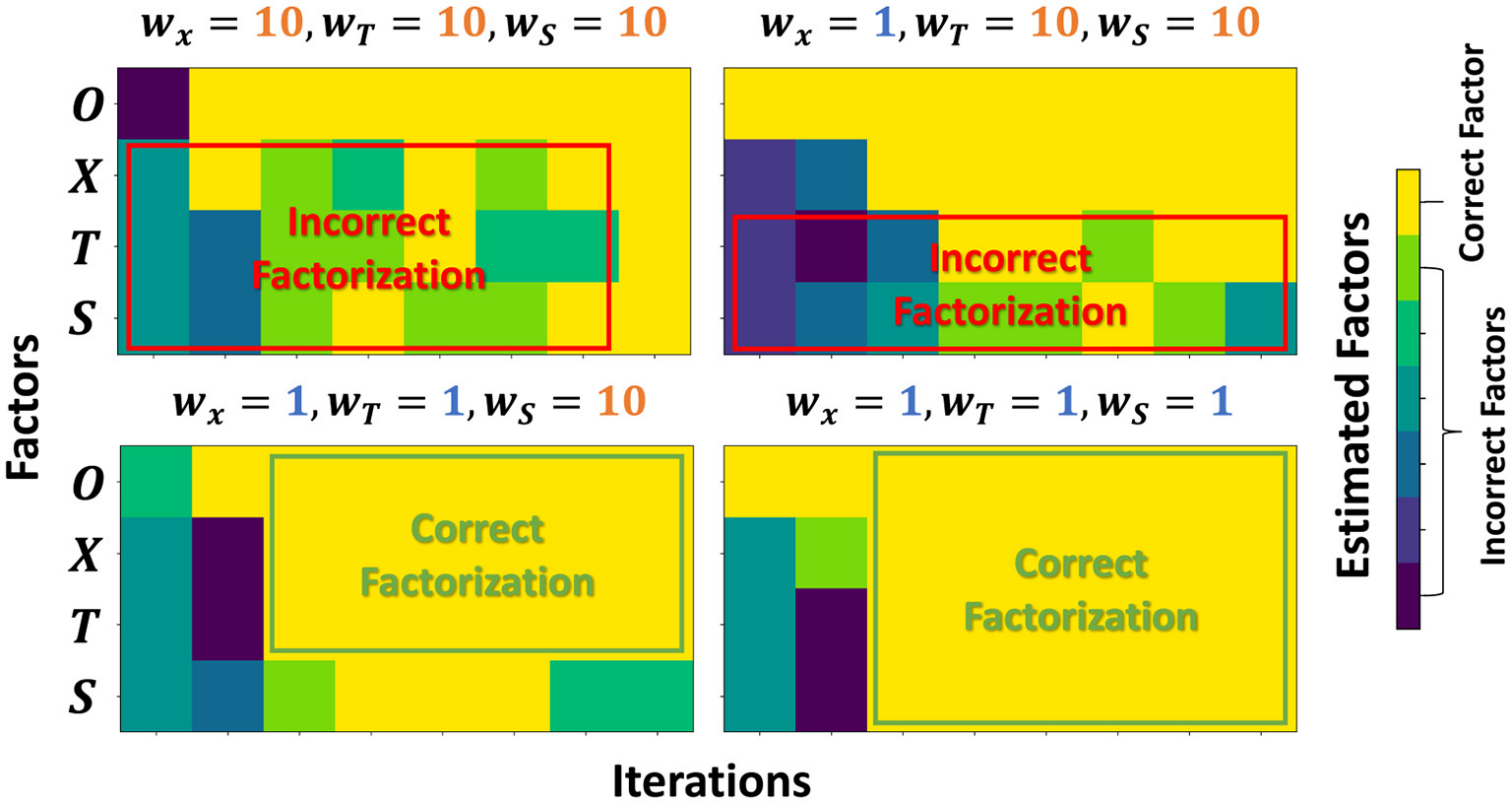
Solution to the resonator network with four factors, with each factor continuously encoded using the random feature encoding.

Finally, we studied factorization via the resonator network in Section 2.6. Objects are sampled from a random codebook O, while position, time, and size are continuously encoded using kernel-generated bases with adjustable length scales *w*_*i*_. We initialized with *w*_*X*_ = *w*_*T*_ = *w*_*S*_ = 10 so that hypervectors for nearby values remain highly correlated. Under this setting, the resonator converges to spurious assignments because the factors are not sufficiently separated in hyperspace. We then sequentially reduced one factor's length scale to *w*_*i*_ = 1 at a time, which decorrelates its codebook and restores identifiability. As shown in Figure 9, setting *w*_*X*_ = 1 enables correct recovery of the position (and the object), while time and size remain ambiguous; subsequently setting *w*_*T*_ = 1 and *w*_*S*_ = 1 resolves the remaining factors. This behavior aligns with the decodability analysis: exclusive (near-orthogonal) encodings minimize cross-terms in the iterative updates and are therefore crucial for reliable multi-factor inference.

## 4 Conclusion

This study examined how kernel-based hyperdimensional encoders mediate a fundamental trade-off between learning and cognition via a single correlation-controlling parameter. Building on complex-valued VSA models, we showed analytically and empirically that the kernel width (*w*_*x*_, *w*_*y*_) imposes opposing requirements across different classes of tasks. Cognitive operations such as decoding and resonator-based factorization benefit from nearly orthogonal positional codes (small *w*), which minimize cross-talk and maximize separability in hyperspace, whereas statistical learning tasks such as classification prefer intermediate *w* values that preserve intra-class correlations without collapsing inter-class structure. We captured these effects using simple separation metrics that quantify the overlap of similarity distributions during decoding and the class-centroid separability during learning.

The observed correlation-orthogonality tradeoff suggests a practical design guideline for future HDC encoders. Rather than treating the encoder as a fixed, task-agnostic module, kernel-based HDC systems can expose *w* (and related parameters) as explicit knobs that are tuned according to the dominant role of the representation. When precise retrieval, error correction, or factorization is paramount, the encoder should favor low-correlation, near-orthogonal hypervectors. When learning and generalization from limited data are central, the encoder should be configured in an intermediate regime where representations remain correlated enough to capture shared structure while still maintaining class-level separation. In this sense, the same underlying encoding mechanism can be adapted across applications by changing only the correlation scale of the positional basis, rather than redesigning the entire HDC pipeline.

A fully systematic, data-driven method for selecting the kernel width *w* across tasks and datasets remains an open problem. Section 3.2 proposed a heuristic link between empirical spatial co-activation statistics and (*w*_*x*_, *w*_*y*_) via estimated correlation lengths, which provides an intuitive initialization rule but is not yet a complete tuning algorithm. In all experiments, we therefore swept *w* over a range of values to map out performance curves, rather than fixing *w* solely from this heuristic. Developing a principled selection strategy (for example, by combining our separation metrics with data-driven correlation estimates or using validation-based model selection) is an important direction for future work.

Our empirical study focused on a toy 5 × 5 dataset and binarized, downsampled MNIST to keep the analysis transparent and to isolate the effects of the kernel width. However, the encoding formulation is linear in the input values *f*_*X, Y*_ and is not limited to binary images. The same approach can, in principle, be applied to real-valued images, time series, and other sensory modalities by replacing binary pixel values with continuous intensities or feature amplitudes in the superposition. Extending the present framework to such continuous-valued and higher-dimensional datasets and to multimodal settings where different channels may require distinct correlation scales is left for future work. We expect that the correlation–orthogonality trade-off characterized here will continue to provide a useful lens for designing task-aware HDC encoders in these more complex domains.

## Data Availability

Publicly available datasets were analyzed in this study. This data can be found here: https://cocodataset.org (Lin et al., 2015).
